# Anti-Atherogenic Properties of *Allium ursinum* Liophylisate: Impact on Lipoprotein Homeostasis and Cardiac Biomarkers in Hypercholesterolemic Rabbits

**DOI:** 10.3390/ijms17081284

**Published:** 2016-08-10

**Authors:** Mariann Bombicz, Daniel Priksz, Balazs Varga, Rudolf Gesztelyi, Attila Kertesz, Peter Lengyel, Peter Balogh, Dezso Csupor, Judit Hohmann, Harjit Pal Bhattoa, David D. Haines, Bela Juhasz

**Affiliations:** 1 Department of Pharmacology, Faculty of Pharmacy, University of Debrecen, Debrecen H-4032, Hungary; bombicz.mariann@pharm.unideb.hu (M.B.); priksz.daniel@pharm.unideb.hu (D.P.); varga.balazs@pharm.unideb.hu (B.V.); gesztelyi.rudolf@pharm.unideb.hu (R.G.); ddhaines2002@yahoo.com (D.D.H.); 2 Institute of Pharmacology and Pharmacotherapy, Faculty of Medicine, University of Debrecen, Debrecen H-4032, Hungary; 3 Department of Cardiology, Faculty of Medicine, University of Debrecen, Debrecen H-4032, Hungary; dr.kertesz.attila@gmail.com; 4 Institute of Applied Informatics and Logistics, University of Debrecen, Debrecen H-4032, Hungary; lengyel.peter@econ.unideb.hu; 5 Department of Research Methodology and Statistics, Institute of Sectoral Economics and Methodology, University of Debrecen, Debrecen H-4032, Hungary; balogh.peter@econ.unideb.hu; 6 Department of Pharmacognosy, Faculty of Pharmacy, University of Szeged, Szeged H-6720, Hungary; csupor.dezso@pharmacognosy.hu (D.C.); hohmann@pharm.u-szeged.hu (J.H.); 7 Department of Laboratory Medicine, Faculty of Medicine, University of Debrecen, Debrecen H-4032, Hungary; harjit@med.unideb.hu

**Keywords:** *Allium* species, atherosclerosis, lipoprotein, cardiovascular homeostasis, echocardiography

## Abstract

The present investigation evaluates the capacity of *Allium ursinum* (wild garlic) leaf lyophilisate (WGLL; alliin content: 0.261%) to mitigate cardiovascular damage in hypercholesterolemic rabbits. New Zealand rabbits were divided into three groups: (i) cholesterol-free rabbit chow (control); (ii) rabbit chow containing 2% cholesterol (hypercholesterolemic, HC); (iii) rabbit chow containing 2% cholesterol + 2% WGLL (hypercholesterolemic treated, HCT); for eight weeks. At the zero- and eight-week time points, echocardiographic measurements were made, along with the determination of basic serum parameters. Following the treatment period, after ischemia-reperfusion injury, hemodynamic parameters were measured using an isolated working heart model. Western blot analyses of heart tissue followed for evaluating protein expression and histochemical study for the atheroma status determination. WGLL treatment mediated increases in fractional shortening; right ventricular function; peak systolic velocity; tricuspidal annular systolic velocity in live animals; along with improved aortic and coronary flow. Western blot analysis revealed WGLL-associated increases in HO-1 protein and decreases in SOD-1 protein production. WGLL-associated decreases were observed in aortic atherosclerotic plaque coverage, plasma ApoB and the activity of LDH and CK (creatine kinase) in plasma. Plasma LDL was also significantly reduced. The results clearly demonstrate that WGLL has complex cardioprotective effects, suggesting future strategies for its use in prevention and therapy for atherosclerotic disorders.

## 1. Introduction

Wild garlic (*Allium ursinum* L.) is a wild plant belonging to the Amaryllidaceae family. It is distributed widely in Asia and Europe and known variously as bear’s garlic, buckrams, bear’s leek, wood garlic and ransoms [1]. The intense flavor of the plant makes it a popular flavoring and regular dietary component for people and animals living in regions where it grows [2]. The mild garlic-like scent of the plant is attributable to its content of sulfur-containing compounds. These include, prominently, sulfoxides and glutamyl peptides. The species also contains high levels of odorless, non-volatile metabolites: *S*-alk(en)yl-l-cysteine-sulfoxides, which hydrolyze under physiological conditions to volatile (poly)sulfides and thiosulfinates, imparting the characteristic odor and flavor of the plant [3]. Wild garlic also contains high levels of polyphenolic compounds, particularly in leaves and bulbs, which accounts substantially for the antioxidant and therapeutic properties of these sections of the plant [4,5,6]. It also combines two additional health-enhancing properties: the plant has approximately 20-times the level of adenosine as common garlic (*Allium sativum*), plus it has significantly higher levels of ajoene, both of which combine to stabilize blood pressure and cholesterol levels, reduce excessive thrombocyte aggregation and improve physiological control of cholesterol metabolism [7]. Indeed, the cardiovascular benefits of using this plant were observed to be so substantial that the Association for the Protection and Research on European Medicinal Plants designated it “Plant of the Year” for 1992 [7]. However, contrary to common garlic, wild garlic has not been studied in clinical trials, and although its cardiovascular effect may be hypothesized based on its chemical constituents, the preclinical confirmation is rather incomplete. *A. ursinum* was also selected as the subject of the present investigation based on the outcomes of previous work by this laboratory demonstrating cardioprotective properties of other plant extracts derived from traditional medicines [8,9].

Hypercholesterolemia, a syndrome characterized by abnormally elevated levels of blood cholesterol and lipoproteins [10,11], was chosen as a model disease for the present study due to its association with a wide range of pathologies, particularly atherosclerosis [12], with associated thrombosis, stroke and heart failure [13]. Although *A. ursinum* is not typically used as a stand-alone medication, its anti-atherogenic properties are well known, to the extent it is used as a dietary treatment for these disorders at Bucharest University Hospital in Romania [14]. In vitro evaluation for the effects of several *A. ursinum* fresh leaf extract preparations on the aggregation of human platelets revealed that ADP-induced aggregation was significantly suppressed by ethanolic extracts. The observed data suggested the similarity of pharmacological action to clopidogrel, a thienopyridine clot formation inhibitor that is a potent antiplatelet drug [15]. A likely explanation for this outcome is the known antiaggregatory properties of the β-sitosterol 3-*O*-β-d-glucopyranoside and 1,2-di-*O*-α-linolenoyl-3-*O*-β-d-galactopyranosyl-sn-glycerol (DLGG) components of *A. ursinum* [16]. It was further noted that 45-day administration of feed supplemented with 1% *w*/*w* wild garlic *Allium ursinum* (wild garlic) or alternatively with 1% *w*/*w* *Allium sativum* (cultivated garlic) to spontaneously hypertensive rats (SHR), in groups of 10 animals per experiment, mediated a significant reduction in final mean systolic blood pressure (SBP) [17].

The possible underlying mechanisms include the ability of ramson to inhibit the activity of angiotensin-converting enzyme (ACE). In vitro tests on the water extract from the leaves (at the concentration of 0.300 mg/mL) showed significantly increased activity on enzyme inhibition when compared to leaves with extract of garlic (58% versus 30%) [7]. Moreover, significantly lower levels of ACE activity were noted in the blood of animals fed for eight weeks with a standard rodent chow containing 2% pulverized whole leaf *A. ursinum*, versus untreated control rats [18].

The physiologic significance of hypercholesterolemia induced by elevated cholesterol in feed administered to animals is particularly well illustrated by the consideration of how such diets affect inflammatory processes, the dysregulation of which imposes increased oxidative stress on a wide range of tissues and to which cells of the cardiovascular system are particularly sensitive [19]. For example, pigs maintained on diets supplemented with 2% cholesterol exhibited impairment of coronary endothelial function associated with decreased capacity to neutralize free radicals, decreased expression of nitric oxide synthetase and elevated activation of nuclear factor-κβ, a pro-inflammatory transcription factor [20]. Outcomes of these investigations underscore the particular significance of hypercholesterolemia for the investigation of cardiac function, as was demonstrated in by the gene transfer studies conducted [21,22].

Previous work by the authors shows that methods of extraction used to recover, purify and concentrate plant products may cause some degradation in the bioactivity of component molecules [23]. For this reason, lyophilization was used to process the *A. ursinum* administered to animals in the present study. This method is easily accomplished and optimally preserves the native properties of extracted biological molecules [24].

## 2. Results

### 2.1. Bioanalytical Analysis of Wild Garlic Leaf Lyophilisate

**Figure 1:**
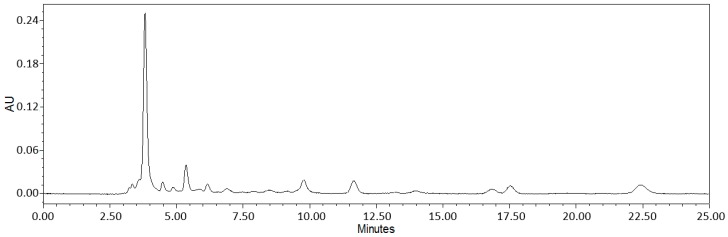
High-performance liquid chromatography (HPLC) spectrum analysis: HPLC chromatogram of the wild garlic leaf lyophilisate (WGLL) reporting one major peak at 3.8 min.

Alliin (*S*-allyl-l-cysteine sulfoxide) is a non-protein amino acid abundant in most of the *Allium* species. It is the natural substrate of alliinase. Therefore, its content in the pure form is commonly analyzed by HPLC. The percentage of total alliin was analyzed by HPLC (Figure 1). Analysis of a representative lyophilized sample revealed the leaf to contain 0.261% alliin by weight (RSD% = 0.45%). The major peak at 3.8 min (detection at 204 nm) is identical to alliin based on its identical UV spectrum and detection time to those of a reference standard.

### 2.2. Echocardiographic Analyses

All echocardiographic examinations were completed within a 20-min time interval with outcomes shown in Table 1. The end-systolic diameter (ESD) of the left ventricle measured in M-mode exhibited significant increases in hypercholesterolemic (HC) animals (1.242 ± 0.045 cm for HC, versus 1.016 ± 0.091 cm noted in the control group). Nevertheless, no change in this outcome was observed in wild garlic leaf lyophilisate (WGLL)-treated hypercholesterolemic (HCT) animals (1.184 ± 0.020 cm) in comparison with this parameter in the control group.

Fractional shortening (FS) and ejection fraction (EF) data correlated strongly with measurements of both the parasternal long and short axis views. FS and EF of HC animals were significantly decreased in comparison with this outcome evaluated in animals in the control group (FS_HC_: 29.010 ± 1.056, versus FS_Control_: 32.310 ± 0.718; and EF_HC_: 49.810 ± 1.140, versus EF_Control_: 56.910 ± 1.294, respectively). Additionally, significant increases in fractional shortening were observed in the HCT group in comparison with the HC group (FS_HCT_: 32.970 ± 1.131 and EF_HCT_: 55.990 ± 1.756). The diastolic function of the left ventricle was evaluated by peak mitral early diastolic inflow velocity/peak atrial diastolic inflow velocity (E/A) ratios measured in Doppler (pulsed wave, PW) mode. E/A ratios were significantly lower in the HC group in comparison to the control animals (HC: 1.207 ± 0.037 versus the control: 1.376 ± 0.045). These results notwithstanding, no significant changes were observed in the E/A ratios of treated animals (HCT: 1.344 ± 0.076) in comparison to controls. Deceleration time of the E wave (DecT) exhibited significant lengthening in the HC animals (HC: 87.440 ± 3.534 ms versus the control: 71.250 ± 4.101 ms). However, DecT values of WGLL-treated animals were significantly lower compared to the HC rabbits (HCT: 69.540 ± 4.787 ms). Tissue velocity imaging (TDI) revealed a non-significant trend toward decreased lateral E’/A’ ratios in WGLL-treated animals. Surprisingly, right ventricle function characterized by measuring peak systolic velocity (S’) waves and tricuspidal annular plane systolic excursion (TAPSE) exhibited significant improvement in WGLL-treated animals. The amplitudes of S’ waves were significantly increased in WGLL-treated animals, compared to the HC group (HCT: 9.156 ± 0.210 cm/s versus HC: 8.103 ± 0.216 cm/s), and TAPSE values were also significantly elevated in the WGLL-treated HCT animals compared to the HC rabbits (HCT: 0.646 ± 0.020 cm versus HC: 0.5762 ± 0.015 cm). Additionally, right ventricle E’/A’ ratios of WGLL-treated animals were slightly decreased.

### 2.3. Cardiac Function in Isolated Working Hearts

**Figure 2:**
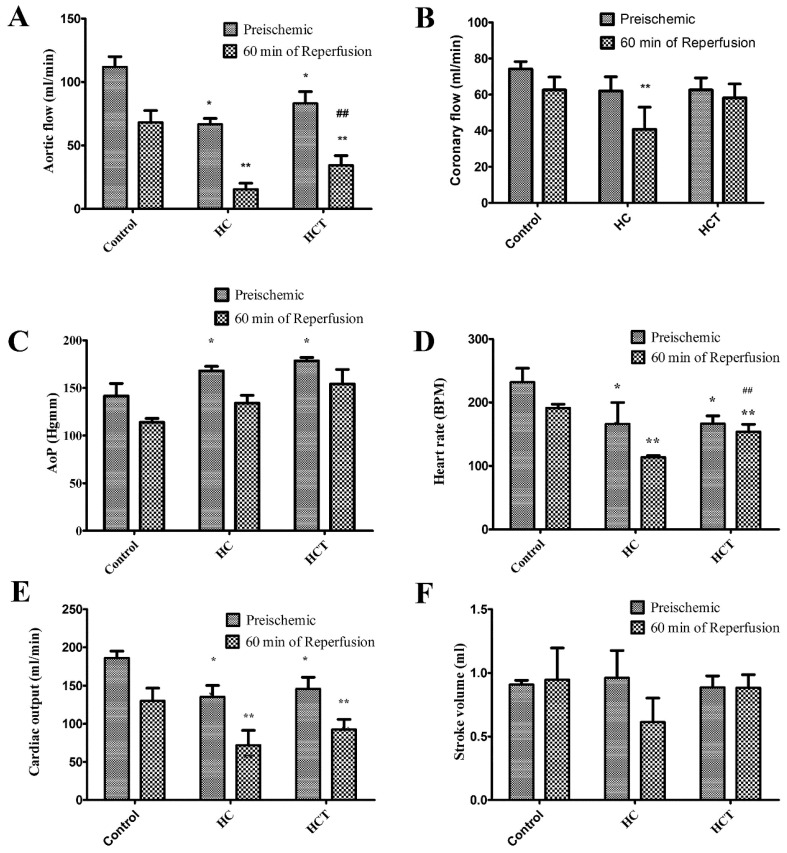
Effect of high cholesterol and WGLL on cardiac function. Hearts were isolated from three groups of animals (*n* = 6), defined as follows: non-hypercholesterolemic animals fed with normal chow (control); hypercholesterolemic group fed with 2% cholesterol-supplemented chow (HC); and a group of hypercholesterolemic animals treated with WGLL (HCT). Isolated working hearts harvested from each animal in each group were subjected to global ischemia followed by 120 min of reperfusion (I/R). Cardiac functions were registered before ischemia (preischemic) and 60 min after global ischemia (60 min of reperfusion). Results are shown as average values from each group of rabbits ± SEM of aortic flow (AF, mL/min, **A**); coronary flow (CF, mL/min, **B**); aortic pressure (AoP, Hgmm, **C**); heart rate (HR, beat/min, **D**); cardiac output (CO, mL/min, **E**); stroke volume (SV, mL, **F**). * *p* < 0.05 compared to the preischemic control; ** *p* < 0.05 compared to the 60 min of reperfusion control; **##** *p* < 0.05 compared to the 60 min of reperfusion HC.

Figure 2 shows the effect on cardiac functional parameters of elevated dietary cholesterol-induced hypercholesterolemia and WGLL treatment. Cardiac functions evaluated included: aortic flow (AF, Figure 2A), coronary flow (CF, Figure 2B), aortic pressure (AoP, Figure 2C), heart rate (HR, Figure 2D), cardiac output (CO, Figure 2E) and stroke volume (SV, Figure 2F). Measurement of these functions in animals in the HC and HCT groups revealed decreases in AF, HR and CO for basal functions of the perfused hearts, compared to controls (*p* < 0.05). There were significant increases under preischemia AoP, both in hypercholesterolemic and WGLL-treated hypercholesterolemic groups, compared to the control group (*p* < 0.05). After 60 min of reperfusion, animals in all groups showed decreases in AF, CF, HR and CO compared to preischemic values. Significant increases in the recovery of AF and HR were observed in the WGLL-treated group, compared to the other groups (*p* < 0.05). Subsequent correlation with the results of echocardiographic measurements (Table 1) further supported the cardioprotective capacity of WGLL.

### 2.4. Western Blot Analysis for Biomarkers of Cardiac Tissue Function

**Figure 3:**
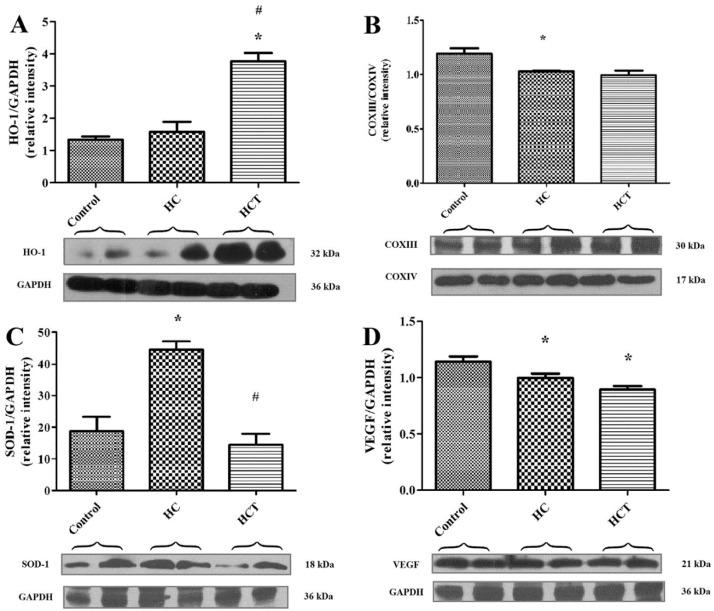
Cardiac tissue biomarker expression: Western blot outcomes. The expression of HO-1 (**A**), COXIII (**B**), SOD-1 (**C**) and VEGF (**D**) protein in rabbit myocardial tissue was measured in homogenized left ventricular cardiac tissue samples drawn from three test groups (*n* = 6), defined as follows: I/R injured hearts from non-hypercholesterolemic animals fed with normal, non-cholesterol supplemented chow (control); I/R injured hearts from hypercholesterolemic animals fed with 2% cholesterol-supplemented chow (HC); and I/R-injured hearts harvested from hypercholesterolemic animals fed with 2% cholesterol and 2% wild garlic leaf lyophilisate-supplemented chow (HCT). GAPDH and COXIV expression levels were measured as reference proteins. Western blot analysis was conducted on each tissue homogenate in duplicate, and the signal intensity of the resulting bands corresponding to proteins of interest was measured using the Scion for Densitometry Image program, Alpha 4.0.2.3. The tissue content of each protein is shown in arbitrary units as the mean for each group of animal ± SEM. * *p* < 0.05 for comparison of the average expression levels of HO-1, COXIII, SOD-1 and VEGF in myocardium to the non-hypercholesterolemic group (control); **#** *p* < 0.05 for comparison to the hypercholesterolemic group (HC).

Myocardial tissue levels of four major mediators of cardiac homeostasis, measured by Western blot analysis, are shown in Figure 3. The outcomes of treatments administered to rabbits in these experiments revealed that expression of HO-1 protein was significantly greater in tissue harvested from HCT animals, compared to the levels observed in the HC group (Figure 3A, *p* < 0.05). Tissue expression of SOD-1 in the HC group was observed to be significantly higher compared to control and HCT animals (Figure 3C, *p* < 0.05). COXIII and VEGF proteins were expressed at lower levels both in HC and HCT groups versus quantities of these proteins found in hearts harvested from the control animals (Figure 3B,D, *p* < 0.05).

### 2.5. Rabbit Aortic Histology

**Figure 4:**
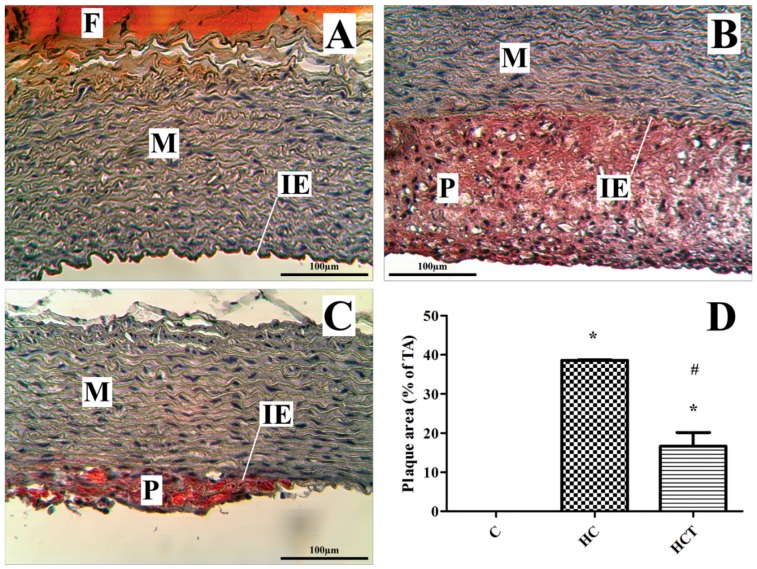
Aortic histologic analysis. Histological sections of aortas from three groups of rabbits stained with hematoxylin-oil red O (**A**–**C**, magnification 25×). Bright red-stained lipid shows atherosclerotic plaques in the HC (**B**) and HCT groups (**C**). Internal elastic lamina is shown as IE; M, media; P, atherosclerotic plaques; and F, adventitial fatty tissue stained with oil red O. Comparison of plaque area in the three groups, expressed as a percentage of the total area (**D**). * *p* < 0.05 for comparison with the control group outcomes; # *p* < 0.05 for comparison with the HC group outcomes.

Histological sections of aortas stained with hematoxylin-oil red O from the three groups are shown in Figure 4. No atherosclerotic lesions were observed in sections of these blood vessels harvested from the control animals fed normal rabbit chow (Figure 4A). At the end of the eight-week treatment period, up to 35% of the total aortic area harvested from HC animals was oil red O positive (Figure 4B). The extent of atherosclerotic lesions observed in animals within the HC group was significantly increased (Figure 4D, 38.610% ± 0.146%) in comparison to lesional coverage in aortic sections taken from animals in the control group (*p* < 0.05). Discrete lesion formation was visualized by oil red O stain and consecutive quantitative analysis in aortas form HCT group rabbits (Figure 4C). Aortas harvested from WGLL-treated animals in the HCT group exhibited significantly reduced atherosclerotic lesional coverage in comparison to the HC group (Figure 4D, 16.710% ± 3.421%, *p* < 0.05).

### 2.6. Serological Correlates of Experimental Treatments

The outcomes of the analyses of peripheral blood serum from animals treated with selected dietary regimens are shown in Table 2. Fasting plasma TC and LDL cholesterol were two orders of magnitude higher, and the HDL cholesterol concentration was eight-fold higher in the HC and six-fold higher in HCT groups, compared to the levels of these analytes in the serum of the control group animals (*p* < 0.05). However, significantly lower plasma TC and LDL cholesterol levels were observed in HCT, versus the HC groups (*p* < 0.05), showing a possible protective effect of the WGLL. Moreover, ApoA levels detected in blood of HC animals (0.022 ± 0.003) were significantly lower, versus those of the control rabbits fed diets with normal cholesterol content (0.042 ± 0.005) and WGLL-treated rabbits in the HCT group (0.056 ± 0.008, *p* < 0.05). No significant differences were noted between serum ApoA content of the serum from animals in the control group, versus the HCT group (*p* > 0.05). Serum levels of ApoB in rabbits from both the HCT and HC groups were significantly higher as compared to the control group (*p* < 0.05). Moreover, ApoB levels detected in the serum of rabbits in the HCT group (0.172 ± 0.019) were significantly lower compared to the content of this analyte in the HC group (0.280 ± 0.063, *p* < 0.05). Additionally, no significant differences were observed between the serum TG content of these three groups. It was further noted that the serum content of the pro-inflammatory biomarker c-reactive protein (CRP) was increased significantly in blood from HC animals, compared to the control group. GOT liver enzyme levels were elevated in the blood of HC group animals (48.8 ± 16.22), as compared to the control and HCT groups (29.670 ± 2.895, 24.910 ± 2.708; *p* < 0.05). LDH (316.6 ± 37.17) and creatine kinase (CK) (2851 ± 600.800) serum levels were significantly decreased in the HCT group compared to the HC group (791.90 ± 325.4 and 4955 ± 1353; *p* < 0.05).

## 3. Discussion

As described in the Results Section 2.1 of this report, bioanalytical analysis of a representative WGLL sample revealed the leaf to contain 0.261% alliin by weight, a property of this material that contributes to its ability to mitigate the expression of other biomolecules described here, which are involved in the atherosclerotic pathophysiologic processes. This natural component of fresh garlic is a sulfoxide derived and formed from the amino acid cysteine [25] and is a major contributor to the capacity of garlic extracts to scavenge hydroxyl radicals, along with a wide variety of other antioxidant properties that counteract oxidative tissue damage [26]. Alliin has also been demonstrated to potently stabilize and strengthen immunoregulation, contributing to the well-known curative properties of garlic [27].

The outcomes of echocardiographic analyses conducted on live animals shown in Table 1 reveal the effect of elevated dietary cholesterol and WGLL treatment. The physiologic significance of these results may be stated according to four major interpretations of the data shown. These may be summarized as follows:

(1) The observed stability throughout the evaluation period of heart rates, respiratory frequencies, M-mode and Doppler measurements demonstrate that basal cardiopulmonary activity was not substantially disrupted by hypercholesteremia, an outcome for which preliminary evidence was provided by an earlier study conducted in the laboratory of the authors [28].

(2) Moreover, in comparison to untreated control rabbits fed a normal diet, the left ventricular end-systolic diameter (ESD) measured in HC animals was significantly increased, with or without WGLL treatment, along with decreased fractional shortening and ejection fraction in HC animals, and a strong correlation was found between fractional shortening (FS) and ejection fraction (EF) data measured on both the parasternal long and short axis views. Furthermore, the effects of WGLL treatment included observations that, relative to HC animals not receiving the lyophilisate, HCT rabbits showed significant increases in fractional shortening. Pathologically-increased ESD is associated with greater risk of mortality in heart disease [29], and decreased FS and ES have recently been implicated as contributors a to fibrotic disease [30]. These results suggest that WGLL will contribute to the survival of cardiac patients and a lower propensity for the development of fibrosis.

(3) Table 1 diastolic function data, generated in Doppler (PW) mode, also reveal that elevated dietary cholesterol resulted in significantly lower left ventricular E/A ratios relative to those observed in control animals. WGLL-treated animals showed values close to controls. Moreover, significant lengthening was observed in the deceleration time of the E wave (DecT) in HC animals, showing an abnormal diastolic filling pattern of the ventricle, which was counteracted by the WGLL-supplemented diet, indicating slightly improved diastolic function.

(4) Finally, surprisingly significant increases were shown in Table 1, in right ventricular function mediated by the WGLL treatment of animals fed elevated cholesterol diets, which were obtained through the evaluation of peak systolic velocity (S’) waves and tricuspidal annular plane systolic excursion (TAPSE). Reduction of peak systolic velocity identifies the presence of right ventricle (RV) dysfunction with high sensitivity. This reduction was significant in HC animals, but was counteracted fully by WGLL treatment, showing that the aforementioned beneficial effects of WGLL supplementation can be seen on right ventricle function, as well. In heart failure patients, the reduction of tricuspidal annular systolic velocity is associated with the severity of RV dysfunction. Surprisingly, TAPSE values in the WGLL-treated group were significantly increased even compared to healthy animals. These findings indicates that diet supplemented with WGLL could have positive effects on right ventricle systolic function, but the relevance of these effects and the underlying mechanisms need further investigations.

The evaluation of cardiac functions in Langendorff-mounted isolated working hearts shown in Figure 2 revealed significantly increased preischemic AoP values in both the hypercholesterolemic and WGLL-treated hypercholesterolemic groups, relative to untreated control rabbits. Furthermore, as shown in Figure 2, ischemic-reperfusion injury-associated decreases in AF, CF, HR and CO, versus preischemic values, along with significantly increased recovery of AF and HR in animals fed the lyophilisate further supported the anti-ischemic properties of WGLL. These effects are consistent with previous studies by the authors, in which interventions that decrease oxidative stress on cardiac tissue dramatically improved recovery from ischemic events [31,32,33]. An interpretation of the significance of these outcomes to the cardioprotective ability of WGLL should be considered in the context of the fact that myocardial ischemic events typically reduce cardiac aortic blood pressure (AoP), along with a reduction in myocardial metabolic requirements, coronary blood flow and left ventricular tension. For these reasons, influences that decrease AoP may be either detrimental or beneficial [34]. Thus, whereas increased preischemic AoP in HC animals indicates that such an increase is pathological for animals maintained on a high cholesterol diet, the failure of WGLL treatment to lower AoP suggests that the extract has negligible effect on this aspect of cardiovascular function.

Data described in Section 2.4 of the Results Section of this article and shown in Figure 3 provide ventricular tissue expression profiles of proteins implicated in pathogenesis and adaptive response to atherosclerotic disease. Western blot analysis of myocardial tissue reveals significantly elevated content of HO-1 protein in tissue harvested from HCT animals, versus that taken from rabbits in the HC group. The heat shock protein HO-1 (hsp-32) is a major antioxidant defense enzyme, which is increased in response to trauma intrinsic to a wide range of diseases, including (and especially) atherosclerotic syndromes [35]. Often, the effects of a disease process overwhelm the protective capacity afforded by endogenous HO-1 expression [36,37,38]. However, its cardioprotective effects may be greatly amplified by the administration of pharmacological inducers, as demonstrated by the authors of the present report [28]. The cytoprotective effects correlating with increased expression of HO-1 are a likely effect of heme degradation by this enzyme to produce bilirubin and carbon monoxide (CO), both of which enhance the healthy function of cardiovascular tissue at the concentration normally produced by HO-1 activity during normal heme clearance.

Therapeutic amplification of HO-1 in these studies was achieved using seed kernel extracts of *Prunus cerasus* (sour cherry). The present investigation demonstrates that lyophilisate of wild garlic leaf also mediates this effect. However, since this plant material also stimulates other protective effects, based on the data shown here, it cannot be determined to what extent WGLL-induced HO-1 expression contributes to the specific cardioprotective outcomes revealed.

SOD1 levels measured by Western blot analysis in myocardial tissue of HC animals after ischemia/reperfusion injury were significantly elevated compared to the controls, while SOD1 expression in WGLL-treated animals was maintained at the normal (control) levels. Both apoptotic signaling and adaptive responses to oxidative stress involve processes for which SOD1 activity is a critical component. This enzyme produces molecular oxygen and hydrogen peroxide (H_2_O_2_) as end metabolites of its main activity, which is to neutralize superoxide [39]. H_2_O_2_ is itself a toxic reactive oxygen species (ROS) and may contribute to ischemia-reperfusion injury of myocardial tissue, through abnormally high apoptotic signaling and oxidative tissue damage in ischemic heart disease [40].

SOD1 is known to have a capacity to limit the detrimental effects of ROS by eliminating O_2_^−^ to produce H_2_O_2_, which is eliminated by glutathione peroxidase or by catalase to harmless H_2_O and O_2_, but on the other hand, with free iron(II), H_2_O_2_ also can form free hydroxyl radicals by Fenton’s reaction (see the graphical abstract). High SOD levels along with considerable amounts of Fe^2+^ are associated with increased production of the highly toxic hydroxyl radical and may even enhance the extent of reperfusion injury [41]. An unbalance between the production of prooxidant H_2_O_2_ (SOD1) and antioxidants, such as glutathione peroxidase and catalase, in the cell might lead to a strengthened production of free radicals, which could lead to serious cellular damage. This is supported by the assessment of SOD activity in the blood of MI patients, which revealed that relative to healthy control individuals, SOD levels in the patient group were significantly higher [42]. This difference likely reflects a normal adaptive response to limit oxidative damage to the myocardium imposed by ischemic (and other) tissue injury.

Western blot analyses conducted in this investigation revealed that COXIII and VEGF, which are both implicated in the pathophysiology of cardiovascular syndromes, were expressed at lower levels, both in HC and HCT groups, versus quantities of these proteins found in hearts harvested from the control animals. In COXIII and VEGF protein levels, there were no significant changes between WGLL-treated and hypercholesterolemic groups. Our results suggest that wild garlic may develop its cardioprotective activity via the heme/HO system and has no effect on COXIII and VEGF proteins.

The extent of atherosclerotic plaque coverage on the intimal surface of hematoxylin-oil red O-stained rabbit aortas reveals negligible plaque on sections harvested from control animals maintained on diets with normal cholesterol content and a lesional extent of approximately 35% in sections from HC rabbits (Figure 4). The significantly reduced lesional coverage observed in WGLL-treated rabbits fed high cholesterol chow (HCT) is an effect also observed in previous work by these authors, in which HO-1 expression increased by adding sour cherry seed kernel extract to rabbit chow. This protected against dietary cholesterol-induced arterial plaque formation [28].

The blood of animals utilized in the present study was evaluated for serum analytes expressed at levels that may be used as diagnostic and therapy effect indicators for cardiovascular disease severity along a wide range of other severe inflammatory syndromes. The outcomes of serum parameter measurements are shown in Table 2. They reveal significantly elevated fasting plasma levels of TC and LDL cholesterol, which were two orders of magnitude higher, and HDL cholesterol concentration, which was eight-fold higher in HC and six-fold higher HCT rabbits versus the controls, effects that are an expected result of high cholesterol diets [43]. However, elevated levels of HDL cholesterol in WGLL-treated rabbits may indicate a cardioprotective property of the lyophilisate in the context of the beneficial effects of HDL. Significantly lower TC and LDL cholesterol levels were observed in WGLL-treated (HCT) animals versus groups fed with high cholesterol chow, but no WGLL (HC), demonstrating that the lyophilisate is protective with respect to the influence of these analytes. ApoA levels in the blood of HC animals were significantly lower versus rabbits fed normal chow (control). Thus, the lack of significant differences in the ApoA content of blood from animals fed normal diets (control) versus the content of this molecule in WGLL-treated rabbits maintained on high cholesterol (HCT) indicated a normalizing effect of the lyophilisate on this outcome. The significance of this result is that ApoA-I deficiency causes both hypertriglyceridemia and increased atherosclerosis in animal models [44], which can be counteracted by a WGLL-supported diet.

Analysis of ApoB revealed that systemic levels of this analyte in rabbits from both HCT and HC groups were significantly higher versus its levels in the blood of animals fed chow with normal cholesterol content (controls). Moreover, ApoB levels detected in blood taken from rabbits in the HCT group were significantly lower in comparison to the content of this analyte in the HC group. This result is well correlated with LDL levels measured in the two groups. This finding was expected since ApoB is the primary apolipoprotein of chylomicrons, VLDL, IDL and LDL particles.

Elevation in serum TG of the HC group was tendentious, but not significant compared to that of the controls. One interpretation is that triglyceride metabolism is unaffected by either influence within the constraints of the present study. Further analysis of blood from each of the three test groups revealed significant elevation of c-reactive protein (CRP) in HC animals versus the controls. Since CRP is a reliable systemic indicator of a wide range of inflammatory pathologies, this result implies that levels of dietary cholesterol administered to animals in this study managed to induce the onset of inflammatory processes. The analysis for serum liver enzyme activities demonstrated significantly elevated GOT in blood of the HC group animals versus the control and HCT groups, suggesting a hepatotoxic effect of elevated dietary cholesterol intake, an effect noted by previous investigators [45]. Finally, the significantly lower levels of LDH and CK observed in HCT animals, versus rabbits maintained on high cholesterol (HC), indicated that the effects of dietary supplementation with WGLL may have beneficial effects on impaired liver function caused by the hypercholesterolemic state. TNFα-induced ICAM-1 mRNA transcription, which has been demonstrated by in vitro studies to suppress the adhesion of monocytes to porcine arterial tissues and HUVECs, was significantly inhibited by treatment with alliin (*S*-allyl-l-cysteine sulfoxide). Moreover, alliin is also protected against the depolarization of mitochondrial membrane potential and overproduction of the superoxide anion, which occur as a downstream effect of TNFα, and may correlate with the suppression of NOX4 mRNA transcription by HUVECs. Additionally, treatment of HUVECs with alliin was also observed to reduce TNF-α-mediated ERK1/2 IjB and IjB (but not p38) phosphorylation. These results provide improved insight into the mechanisms by which alliin acts as a countermeasure to atherosclerotic pathomechanisms [46] and are consistent with the protective effects of the plant extract reported here.

## 4. Experimental Section (Methodology)

### 4.1. Sample Lyophilization and Bioanalytics

Deep-frozen *Allium ursinum* leaves (Toltelekgyar Ltd., Zalakomar, Hungary) were lyophilized for 24 h in a Martin-Christ ALPHA 1-4 freeze dryer (Martin Christ Gefriertrocknungsanlagen GmbH, Osterode am Harz, Germany) at an ambient pressure of 0.120 millibars (mb), with a condensor temperature of −50 °C and shelf temperature of 35 °C. The ratio of the frozen leaf to fresh and desiccated plant material was 5:6:1. HPLC analysis was accomplished using a Waters 600 system (Waters Corporation, Milford, CT, USA), equipped with a 2998 photodiode array detector, on-line degasser and auto sampler, using a reversed phase Phenomenex Synergi 4 μm Hydro-RP 80Å (250 mm × 4.6 mm) column (Phenomenex, Torrance, CA, USA). With a column temperature of 25 °C, a gradient elution was applied as follows: 0–15 min: 100% of Mobile Phase A (water + 0.1% phosphoric acid); 15–20 min: the ratio of Mobile Phase B (acetonitrile) increased to 100%; 20–25 min: 100% B; 25–27 min: A increased to 100%; 27–45 min: 100% A. The flow rate was 0.75 mL/min, and alliin was monitored at 204 nm. Alliin was detected at 4.6 min. Data acquisition and evaluation were performed using Empower Pro software.

Alliin was purchased from LGC Standards. Acetonitrile used for chromatographic analysis (LiChrosolv^®^ HPLC grade) was obtained from Merck (Merck Consumer Health Holding GmbH, Darmstadt, Germany). The Millipore Direct-Q UV3 clarifier (Merck Millipore, Molsheim, France) was used to produce purified water for HPLC measurements. Stock standard solutions of alliin were prepared with methanol and stored at 4 °C. The calibration range was 0.5–5 µg alliin/injection. The calibration standard was injected in triplicate at six volume levels. Extraction of alliin from the lyophilized plant material was carried out with 10 mL MeOH at room temperature for 3 min from a 1-g sample in an ultrasonic bath. After filtering through a filter membrane (Acrodisc^®^ GHP 13 mm, 0.45 μm, Waters Corporation, Milford, CT, USA), 3 independently-prepared samples were analyzed in triplicate.

### 4.2. Animals

The experiments were carried out using adult male New Zealand rabbits with an average body weight of 2.5–3.0 kg. The animals received human care in compliance with the “Principles of Laboratory Animal Care” by EU Directive 2010/63/EU for animal experiments. The duration of the adaptive feeding was 2 weeks. The rabbits were provided with laboratory rodent chow, or chow enriched with 2.0% cholesterol (Jurasko Ltd., Debrecen, Hungary), or cholesterol-supplemented chow containing 2% wild garlic leaf lyophilisate (WGLL) daily for 8 weeks ad libitum. *Allium ursinum* lyophilisate-containing chow was produced in the laboratory of Department of Pharmaceutical Technology, University of Debrecen. The comparison of the behavior and general health status of animals provided with unsupplemented rabbit chow versus feed containing other components showed no observable differences, with no indication that administration of feed acted as a confounder to the experiments conducted.

### 4.3. Echocardiography

**Figure 5:**
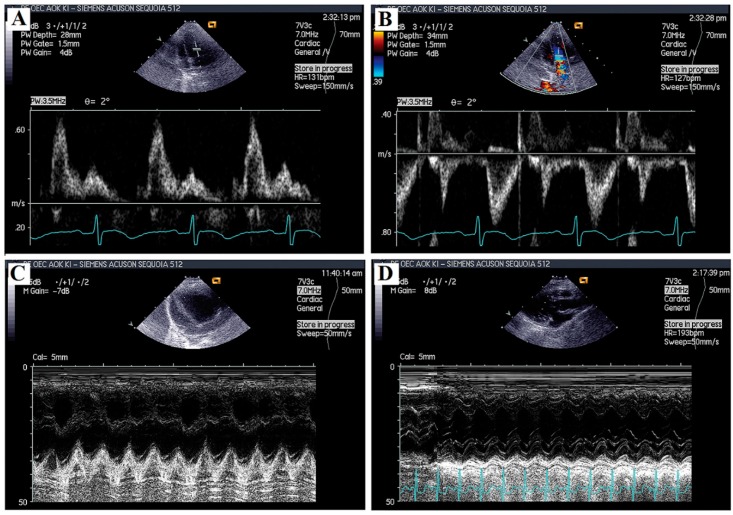
Representative images of echocardiographic measurements. (**A**) Doppler image, mitral inflow velocities; (**B**) color Doppler image, velocity of left ventricle outflow tract; (**C**) M-mode image, tricuspidal annular plane systolic excursion (TAPSE); (**D**) M-mode image, parasternal long axis view (PLAX) of left ventricle.

Echocardiographic examination of animals was conducted under light anesthesia (ketamine 15 mg/kg, xylazine 3 mg/kg (i.m.)) at the 8-week time point of the study [47]. The chest of each animal was shaved, and the rabbit was positioned in a dorsal decubitus position. Echocardiographic measurements were performed using a Siemens Acuson 512 sonograph (Siemens Healthcare GmbH, Erlangen, Germany), with a 7V3c probe at 7 MHz, with fundamental imaging (Figure 5). Conventional measurements were carried out in 2D and M-mode. Parasternal long axis views were obtained and recorded to ensure that the mitral and aortic valves, as well as the apex, were visualized. The parasternal short axis views were recorded at the mid-papillary muscle level. M-mode tracings were performed at the mid-papillary muscle level, either in parasternal long or short axis views. M-mode for visualization and quantification of wall motion in cardiovascular research was used; single line acquisition allows for the very high-temporal (1000 fps) resolution necessary for the analysis of LV function. Echocardiographic measurements included interventricular and left ventricular free-wall thickness in diastole and systole (IVSs, IVSd) and left ventricular internal diameter at end-diastole (LVIDd) and end-systole (LVIDs). End-systolic volume (ESV), end-diastolic volume (EDV), stroke volume (SV) and left ventricular mass were calculated. Fractional shortening was computed by using the equation FS = [(LVIDd − LVIDs)/LVIDd] × 100%, and the ejection fraction was calculated by using the following formula (Teicholz): EF = (LVEDD^2^ − LVESD^2^)/LVEDD^2^. In this latter formula LVEDD means Left Ventricular End Diastolic Dimension, while LVESD is Left Ventricular End Sistolic Dimension. Mitral and tricuspid annular peak systolic excursions (MAPSE and TAPSE) were assessed with M-mode, measuring the distance of mitral or tricuspid annular movement between end-diastole to end-systole. All measurements were averaged over three to five consecutive cardiac cycles.

Doppler (PW) imaging of the mitral valve and aortic valve was obtained from the apical 4-chamber view and the apical 5-chamber view. From the mitral inflow velocity image, the following measurements were obtained: peak E and peak A waves (mitral early and late filling velocities), the E to A ratio (E/A) and deceleration time of early filling velocities (DecT). Aortic and left ventricular outflow tract (LVOT) parameters were also calculated: LVOTVmax, LVOT maxPG and LVOTEnvTi.

Tissue velocity imaging (TVI) measurements were analyzed from the apical 4-chamber view and from the parasternal long axis and short axis views, as well. A 5-mm tissue sampling volume was obtained at the mitral annulus from both septal and lateral walls. From the acquired images, the following functional parameters were measured: S’, E’/A’ wave velocities, E/E’ (early diastolic mitral inflow velocity divided by average value of lateral and septal tissue Doppler early diastolic velocities) and E’/A’ (tissue Doppler early and late diastolic velocity ratio) [48].

### 4.4. Measurement of Serum Parameters

Blood samples were collected with EDTA-K2 evacuated tubes (BD Vacutainer, Becton Dickinson and Company, Franklin Lakes, NJ, USA) from the marginal ear vein of the animals, at the endpoint of the treatment. The samples were collected and processed aseptically to minimize hemolytic activity. The serum level of total cholesterol (TC), triglycerides (TG), high density lipoprotein cholesterol (HDL-C), low density lipoprotein cholesterol (LDL-C) and the value of apolipoprotein A-I (ApoA), apolipoprotein B (ApoB), c-reactive protein (CRP), aspartate transaminase (also called glutamic oxaloacetic transaminase, GOT), lactate dehydrogenase (LDH) and creatine kinase (CK) were detected by automated analyzers in the Department of Laboratory Medicine at the University of Debrecen.

### 4.5. Isolated Working Heart Preparation (Langendorff Method)

Each of the animals was anaesthetized with a mixture of ketamine/xylazine (50/5 mg/kg, intramuscularly). A bolus of heparin was administered (1000 U/kg of body weight, intravenously) 20 min before thoracotomy, to avoid thrombosis. Next, the chest cavity was opened and the pericardium incised. The heart was excised and immediately transferred to ice-cold modified Krebs–Henseleit (mKH) buffer (pH 7.4 on 37 °C, gassed with 95% O_2_ and 5% CO_2_ mixture) [49]. The aorta was then cannulated and the heart perfused for 10 min, retrogradely in Langendorff mode with mKH buffer to clear residual blood from each harvested organ. The perfusate had the following composition: NaCl, 118 mmol/L; NaHCO_3_, 25 mmol/L; KCl, 4.8 mmol/L; CaCl_2_, 1.8 mmol/L; Mg_2_SO_4_, 1.2 mmol/L; KH_2_PO_4_, 1.2 mmol/L; and 10 mM glucose. A dual-headed peristaltic pump controlled the rate of perfusion of mKH buffer. The left atrial appendage was incised, and the pulmonary veins were ligated. A small incision was made at the bifurcation of pulmonary arteries; thus all coronary effluent was collected by the pulmonary artery. Next, the circulation was switched to anterograde perfusion, in order to set the baseline parameters in working heart mode for 20 min.

The following parameters were recorded and the resulting data analyzed using a pressure transducer attached to the aortic outflow line: aortic pressure (AoP), heart rate (beats/min), left ventricular developed pressure (LVDP). Aortic flow (AF, mL/min) and coronary flow (CF, mL/min) were measured by using a flowmeter. Hearts were then subjected to 30 min of global ischemia, then perfused for 15 min in Langendorff mode and converted to working heart mode for 105 min. The above-listed outcomes were measured and recorded during the reperfusion at the 30-, 60-, 90- and 120-min time points. Immediately following 120 min of reperfusion, small myocardial biopsies from LV heart tissue were removed and frozen for subsequent molecular biological analysis.

### 4.6. Histological Analysis of the Aortic Root

Lipid staining was carried out with oil red O (Sigma Diagnostics, St. Louis, MO, USA) by use of the following protocol: aortic tissues were frozen in Optimal Cutting Temperature (OCT) medium (Thermo Fisher Scientific Inc., Waltham, MA, USA). Cryostated tissue sections were cut to a thickness of 6.0 µm and applied to Superfrost Plus slides (Daiggers, Vernon Hills, IL, USA). Atherosclerotic lesions in the aortic root were examined at 3 locations and each separated by 120 μm. Four to 5 serial sections were prepared from each location, starting beyond the aortic arch. The sections were stained, as described previously, with oil red O, followed by analysis of the lipid composition of the lesions, by calculating the percentage of oil red O-positive area, relative to the total cross-sectional vessel wall area. Nuclei were counterstained with hematoxylin (Sigma Diagnostics), using routine methods. All images were captured with a binocular light microscope (Carl Zeiss Microscopy GmbH, Jena, Germany) equipped with a video camera and analyzed using Scion Image software (Scion Corp., Torrance, CA, USA).

### 4.7. Extraction of Myocardial Protein

Three hundred milligrams of frozen tissues from rabbit left ventricular myocardium were homogenized in 800 µL Buffer A (25 mM Tris-HCl, pH 8, 25 mM NaCl, 4 mM Na-orthovanadate, 10 mM NaF, 10 mM Na-pyrophosphate, 10 nM okadaic acid, 0.5 mM EDTA, 1 mM PMSF and protease inhibitor cocktail (Sigma-Aldrich, St. Louis, MO, USA)) in a Polytron-homogenizer. Homogenates were centrifuged at 2000 rpm at 4 °C for 10 min. Supernatant from the above centrifugation was further centrifuged at 10,000 rpm at 4 °C for 20 min, and the resulting supernatant was used as the cytosolic extract. The nuclear pellet was resuspended in 400 µL of Buffer A with 0.1% Triton-X-100 and 500 mM NaCl, then lysed by incubation for one hour on ice. Homogenates were then centrifuged at 14,000 rpm at 4 °C for 10 min, and the supernatant was used as a mitochondrial lysate. Cytosolic mitochondrial extracts were aliquoted, snap frozen and stored at −80 °C for further investigations. The total protein concentration was assayed using the bicinchoninic acid (BCA) method with bovine serum albumin as the standard (Pierce, Rockford, IL, USA).

### 4.8. Western Blot Assays for Protein Expression in Cardiac Tissue

Western blot analysis was used to evaluate left ventricular myocardial tissue for the expression level of the following proteins: heme-oxygenase 1 (HO-1), superoxide-dismutase 1 (SOD1), vascular endothelial growth factor A (VEGF), cytochrome c oxidase III (COXIII), cytochrome c oxidase IV (COXIV) and glyceraldehyde 3-phosphate dehydrogenase (GAPDH). The total protein concentration in cytosolic and mitochondrial extract was determined using the BCA Protein Assay Kit. Next, the protein was diluted with Laemmli buffer and heated to 100 °C for 10 min. The denaturated samples were separated by SDS/polyacrylamide gel electrophoresis (SDS-PAGE) at 120 V for 90 min and transferred onto a nitrocellulose membrane (Bio-Rad Laboratories, Hercules, CA, USA) at 100 V for 1 h. Precision plus Protein Kaleidoscope standards (Bio-Rad Laboratories) were used as molecular-weight standards. The membranes were blocked in 5% low fat milk blocking buffer for 90 min and then incubated overnight at 4 °C with primary antibodies (Sigma-Aldrich). After being washed with Tris-buffered saline containing Tween 20 (TBS-T) three times for 10 min, the membranes were incubated with horseradish peroxidase-labeled secondary antibody diluted 1:2000 in TBS-T and 1% (wt/vol) nonfat dry milk for 90 min at room temperature. Enhanced chemiluminescent substrate (ECL, Litmus Scientific, Advansta Inc., Menlo Park, CA, USA) was used to identify bands. Detection was made by autoradiography for variable lengths of time with Medical X-Ray Film (Agfa-Gevaert N.V., Mortsel, Belgium). Quantitative analysis of scanned blots was carried out using the Scion for Windows Densitometry Image program Version Alpha 4.0.3.2 (Scion Corporation, Frederick, MD, USA). Signal intensity for bands corresponding to each protein of interest was estimated and reported in arbitrary units ± SEM.

### 4.9. Statistical Analysis

All data are presented as the average magnitudes of each outcome in a group ± standard error of the mean (SEM). Statistical analysis was performed using one-way analysis of variance (ANOVA) followed by Kruskal–Wallis multiple comparison tests with GraphPad Prism software for Windows (GraphPad Software Inc., La Jolla, CA, USA). Probability values (*p*) less than 0.05 were considered statistically significant.

## 5. Conclusions

Outcomes of the present report demonstrate that wild garlic leaf lyophilisate improves cardiac functions in isolated hearts harvested from WGLL-treated rabbits. The improvements observed include significantly better post-ischemic values of aortic flow in treated animals compared to the HC group (*p* < 0.05). Coronary flow measurements showed similar trends. Echocardiographic measurements showed improved diastolic heart functions in animals that ate an *Allium ursinum* lyophilisate-containing high-cholesterol diet. Impaired relaxation expressed as DecT and E/A ratios was found in HC animals, while parameters measured in WGLL-treated animals reached normal values. Systolic function expressed as FS and EF was also found significantly decreased in HC animals, and the process was greatly counteracted by WGLL treatment. Interestingly, better right ventricle functions were measured in treated animals (higher peak E-wave velocity and higher TAPSE values). Tissue staining showed significantly decreased atherosclerotic plaque formation in animals treated with HCT compared to the HC group. Total blood cholesterol levels in animals fed with 2% cholesterol-containing diet showed a dramatic increase after the eight-week period, while the values of the control group remain in the physiologic range. Cholesterol levels in animals treated with *Allium ursinum* lyophilisate were significantly lower compared to the HC group (*p* < 0.05). WGLL also had notable beneficial effects on the other monitored serum parameters (GOT, LDH, CK). Important novelties of this present report include the findings that increased dietary cholesterol intake may raise the level of SOD1 in cardiac tissue, which is associated with increased ROS-dependent tissue damage, and this may be counteracted by WGLL treatment; furthermore, our work revealed that WGLL supplementation could elevate the activity of the HO-1-mediated cardioprotective pathway in hypercholesterolemic circumstances.

## Figures and Tables

**Table 1:** Echocardiographic outcomes. Outcomes of echocardiographic evaluations on animals fed normal cholesterol-free rabbit chow (control); normal rabbit chow, containing 2% cholesterol (hypercholesterolemic, HC); rabbit chow containing 2% cholesterol + 2% WGLL (hypercholesterolemic treated, HCT). Outcomes evaluated included the following: heart rate (HR); beats per minute (bpm); aortic diameter (Ao); left ventricle (LV); right ventricle (RV); end-systolic diameter (ESD); end-diastolic diameter (EDD); parasternal long axis view (PLAX); short axis view (SAX); fractional shortening (FS) of the left ventricle; ejection fraction of the left ventricle (EF); calculated weight of the left ventricle (LV mass); peak mitral early diastolic inflow velocity/peak atrial diastolic inflow velocity (E/A); deceleration time of the E wave from maximum to baseline (DecT); peak mitral inflow velocity/average of spectral tissue Doppler peak early diastolic velocities at the septal and lateral corner of mitral annulus (E/E’); maximal velocity of left ventricle outflow (LVOTVmax); left ventricle outflow tract velocity time integral (LVOTVTI); peak early diastolic velocity of the lateral wall, spectral tissue Doppler/peak atrial diastolic velocity of the lateral wall, spectral tissue Doppler (E’/A’); peak systolic velocity (S); mitral annular plane systolic excursion (MAPSE); and tricuspidal annular plane systolic excursion (TAPSE).

Mean ± SEM	HR (bpm)	Ao (cm)	LV ESD (cm)	LV EDD (cm)	FS PLAX (%)
**Control**	180.8 ± 4.145	0.946 ± 0.024	1.016 ± 0.091	1.655 ± 0.050	39.370 ± 5.021
**HC**	150.2 ± 4.303 *	0.919 ± 0.025	1.242 ± 0.045 *	1.756 ± 0.063	29.220 ± 0.803 *
**HCT**	185.0 ± 7.053 **	0.898 ± 0.012	1.184 ± 0.020	1.793 ± 0.031	33.820 ± 1.312 **
	**EF PLAX (%)**	**LV mass PLAX (g)**	**FS SAX (%)**	**EF SAX (%)**	**LV mass SAX (g)**
**Control**	56.910 ± 1.294	6.632 ± 0.478	32.310 ± 0.717	54.130 ± 0.961	6.573 ± 0.351
**HC**	49.810 ± 1.140 *	8.218 ± 0.628	29.010 ± 1.056 *	49.430 ± 1.517 *	8.315 ± 0.792 *
**HCT**	55.990 ± 1.756 **	8.769 ± 0.169 *	32.970 ± 1.131 **	54.930 ± 1.522 **	8.195 ± 0.226 *
	**E/A**	**DecT (ms)**	**E/E’**	**LVOTVmax (cm/s)**	**LVOTVTI (cm)**
**Control**	1.376 ± 0.045	71.250 ± 4.101	1.417 ± 0.058	84.280 ± 2.131	0.071 ± 0.002
**HC**	1.207 ± 0.037 *	87.440 ± 3.534 *	1.775 ± 0.101	87.940 ± 5.719	0.080 ± 0.005
**HCT**	1.344 ± 0.076	69.540 ± 4.787 **	1.718 ± 0.155	77.150 ± 2.157	0.0685 ± 0.002
	**E’/A’ (lateral)**	**MAPSE (cm)**	**RV S′ (cm/s)**	**RV E′/A′**	**TAPSE (cm)**
**Control**	1.303 ± 0.058	0.527 ± 0.018	8.935 ± 0.273	1.336 ± 0.051	0.576 ± 0.012
**HC**	1.109 ± 0.071	0.571 ± 0.025	8.103 ± 0.215 *	1.233 ± 0.092	0.576 ± 0.015
**HCT**	1.065 ± 0.117	0.592 ± 0.030 *	9.156 ± 0.210 **	1.055 ± 0.077 *	0.644 ± 0.020 **

* *p* < 0.05 in comparison to mean the values of the control group; ** *p* < 0.05 in comparison to the mean values of the HC group.

**Table 2:** Serum biomarkers of cardiac function. Average serum TC, TG, LDL-C, HDL-C (mmol/L), ApoA and ApoB (g/L), CRP (mg/L), GOT, LDH and CK (U/L) levels in 3 groups of rabbits (*n* = 6), were analyzed by an automated hematology analyzer. Each analysis was conducted on peripheral blood serum harvested from animals following the 8-week treatment periods, for: non-hypercholesterolemic (control), hypercholesterolemic rabbits (HC) and hypercholesterolemic animals receiving WGLL-supplemented chow (HCT). Results are shown as the average values from each group of animals ± SEM of serum total cholesterol (serum cholesterol, mmol/L) and low-density lipoprotein cholesterol (LDL-C, mmol/L).

Groups	TC	LDL-C	HDL-C	ApoA	ApoB
**Control**	0.793 ± 0.067	0.230 ± 0.023	0.523 ± 0.045	0.042 ± 0.005	0.015 ± 0.003
**HC**	26.370 ± 3.660 *^,#^	23.550 ± 3.032 *^,#^	4.427 ± 0.656 *	0.022 ± 0.003 *^,#^	0.280 ± 0.063 *^,#^
**HCT**	20.030 ± 1.947 *^,^**	17 ± 1.942 *^,^**	3.314 ± 0.369 *	0.056 ± 0.008 **	0.172 ± 0.019 *^,^**
	**TG**	**CRP**	**GOT**	**LDH**	**CK**
**Control**	0.788 ± 0.035	0.100 ± 0.014	29.670 ± 2.895	726.400 ± 170.700	4213 ± 623.200
**HC**	1.367 ± 0.335	0.728 ± 0.362 *	48.800 ± 16.220 *	791.900 ± 325.400	4955 ± 1353
**HCT**	1.052 ± 0.339	0.596 ± 0.231	24.910 ± 2.708 **	316.600 ± 37.170 **	2851 ± 600.800 **

* *p* < 0.05 compared to the control group values; ** *p* < 0.05 compared to the HC group values; # *p* < 0.05 compared to the HCT group values.

## References

[1] Kilian R., Hanelt P., Research I.P.G.C.P., Kilian W. (2001). Mansfeld’s Encyclopedia of Agricultural and Horticultural Crops: Except Ornamentals.

[2] Assessment G.F.I.F.R. Risk of mix-up with bear’s garlic. http://www.bfr.bund.de/en/press_information/2005/10/risk_of_mix_up_with_bears_garlic-6228.html.

[3] Yoo K., Pike L. (1998). Determination of flavor precursor compound s-alk(en)yl-l-cysteine sulfoxides by an HPLC method and their distribution in *Allium* species. Sci. Hortic..

[4] Djurdjevic L., Dinic A., Pavlovic P., Mitrovic M., Karadzic B., Tesevic V. (2004). Allelopathic potential of *Allium ursinum* L.. Biochem. Syst. Ecol..

[5] Gitin L., Dinica R., Parnavel R. (2012). The influence of extraction method on the apparent content of bioactive compounds in romanian *Allium* spp. Leaves. Not. Bot. Horti Agrobot. Cluj-Napoca.

[6] Gođevac D., Vujisić L., Mojović M., Ignjatović A., Spasojević I., Vajs V. (2008). Evaluation of antioxidant capacity of *Allium ursinum* L. Volatile oil and its effect on membrane fluidity. Food Chem..

[7] Sendl A., Elbl G., Steinke B., Redl K., Breu W., Wagner H. (1992). Comparative pharmacological investigations of *Allium ursinum* and *Allium sativum*. Planta Med..

[8] Bak I., Lekli I., Juhasz B., Nagy N., Varga E., Varadi J., Gesztelyi R., Szabo G., Szendrei L., Bacskay I. (2006). Cardioprotective mechanisms of prunus cerasus (sour cherry) seed extract against ischemia-reperfusion-induced damage in isolated rat hearts. Am. J. Physiol.. Heart Circ. Physiol..

[9] Lekli I., Szabo G., Juhasz B., Das S., Das M., Varga E., Szendrei L., Gesztelyi R., Varadi J., Bak I. (2008). Protective mechanisms of resveratrol against ischemia-reperfusion-induced damage in hearts obtained from zucker obese rats: The role of glut-4 and endothelin. Am. J. Physiol. Heart Circ. Physiol..

[10] Durrington P. (2003). Dyslipidaemia. Lancet.

[11] Posa A., Szabo R., Kupai K., Csonka A., Szalai Z., Veszelka M., Torok S., Daruka L., Varga C. (2015). Exercise training and calorie restriction influence the metabolic parameters in ovariectomized female rats. Oxid. Med. Cell. Longev..

[12] Bhatnagar D., Soran H., Durrington P.N. (2008). Hypercholesterolaemia and its management. BMJ.

[13] Finn A.V., Nakano M., Narula J., Kolodgie F.D., Virmani R. (2010). Concept of vulnerable/unstable plaque. Arterioscler. Thromb. Vasc. Biol..

[14] Epure L.I., Roman G.V., Mărăcineanu R. (2011). Studies on medicinal and aromatic plants used in the therapeutic recipes in the bucharest university hospital. Sci. Pap. Univ. Agron. Sci. Vet. Med. Buchar. Ser. A Agron..

[15] Hiyasat B., Sabha D., Grotzinger K., Kempfert J., Rauwald J.W., Mohr F.W., Dhein S. (2009). Antiplatelet activity of *Allium ursinum* and *Allium sativum*. Pharmacology.

[16] Sabha D., Hiyasat B., Grotzinger K., Hennig L., Schlegel F., Mohr F.W., Rauwald H.W., Dhein S. (2012). *Allium ursinum* L.: Bioassay-guided isolation and identification of a galactolipid and a phytosterol exerting antiaggregatory effects. Pharmacology.

[17] Preuss H.G., Clouatre D., Mohamadi A., Jarrell S.T. (2001). Wild garlic has a greater effect than regular garlic on blood pressure and blood chemistries of rats. Int. Urol. Nephrol..

[18] Rietz B., Isensee H., Strobach H., Makdessi S., Jacob R. (1993). Cardioprotective actions of wild garlic (*Allium ursinum*) in ischemia and reperfusion. Mol. Cell. Biochem..

[19] Haines D.D., Juhasz B., Tosaki A. (2013). Management of multicellular senescence and oxidative stress. J. Cell. Mol. Med..

[20] Rodriguez-Porcel M., Lerman L.O., Holmes D.R., Richardson D., Napoli C., Lerman A. (2002). Chronic antioxidant supplementation attenuates nuclear factor-κβ activation and preserves endothelial function in hypercholesterolemic pigs. Cardiovasc. Res..

[21] Gordts S.C., van Craeyveld E., Muthuramu I., Singh N., Jacobs F., de Geest B. (2012). Lipid lowering and HDL raising gene transfer increase endothelial progenitor cells, enhance myocardial vascularity, and improve diastolic function. PLoS ONE.

[22] Van Craeyveld E., Jacobs F., Gordts S.C., de Geest B. (2012). Low-density lipoprotein receptor gene transfer in hypercholesterolemic mice improves cardiac function after myocardial infarction. Gene Ther..

[23] Iloki-Assanga S.B., Lewis-Lujan L.M., Lara-Espinoza C.L., Gil-Salido A.A., Fernandez-Angulo D., Rubio-Pino J.L., Haines D.D. (2015). Solvent effects on phytochemical constituent profiles and antioxidant activities, using four different extraction formulations for analysis of bucida buceras l. And phoradendron californicum. BMC Res. Notes.

[24] Halpern G.M. (2000). Anti-inflammatory effects of a stabilized lipid extract of *Perna canaliculus* (Lyprinol^®^). Allerg. Immunol..

[25] Rybak M.E., Calvey E.M., Harnly J.M. (2004). Quantitative determination of allicin in garlic: Supercritical fluid extraction and standard addition of alliin. J. Agric. Food Chem..

[26] Ariga T., Seki T. (2006). Antithrombotic and anticancer effects of garlic-derived sulfur compounds: A review. BioFactors.

[27] Salman H., Bergman M., Bessler H., Punsky I., Djaldetti M. (1999). Effect of a garlic derivative (alliin) on peripheral blood cell immune responses. Int. J. Immunopharmacol..

[28] Juhasz B., Kertesz A., Balla J., Balla G., Szabo Z., Bombicz M., Priksz D., Gesztelyi R., Varga B., Haines D.D. (2013). Cardioprotective effects of sour cherry seed extract (SCSE) on the hypercholesterolemic rabbit heart. Curr. Pharm. Des..

[29] Tribouilloy C., Grigioni F., Avierinos J.F., Barbieri A., Rusinaru D., Szymanski C., Ferlito M., Tafanelli L., Bursi F., Trojette F. (2009). Survival implication of left ventricular end-systolic diameter in mitral regurgitation due to flail leaflets a long-term follow-up multicenter study. J. Am. Coll. Cardiol..

[30] Carluccio E., Biagioli P., Zuchi C., Bardelli G., Murrone A., Lauciello R., D’Addario S., Mengoni A., Alunni G., Ambrosio G. (2016). Fibrosis assessment by integrated backscatter and its relationship with longitudinal deformation and diastolic function in heart failure with preserved ejection fraction. Int. J. Cardiovasc. Imaging.

[31] Csepanyi E., Czompa A., Haines D., Lekli I., Bakondi E., Balla G., Tosaki A., Bak I. (2015). Cardiovascular effects of low versus high-dose beta-carotene in a rat model. Pharmacol. Res..

[32] Czompa A., Gyongyosi A., Czegledi A., Csepanyi E., Bak I., Haines D.D., Tosaki A., Lekli I. (2014). Cardioprotection afforded by sour cherry seed kernel: The role of heme oxygenase-1. J. Cardiovasc. Pharmacol..

[33] Haines D.D., Bak I., Ferdinandy P., Mahmoud F.F., Al-Harbi S.A., Blasig I.E., Tosaki A. (2000). Cardioprotective effects of the calcineurin inhibitor FK506 and the PAF receptor antagonist and free radical scavenger, EGb 761, in isolated ischemic/reperfused rat hearts. J. Cardiovasc. Pharmacol..

[34] Isoyama S., Maruyama Y., Ashikawa K., Sato S., Suzuki H., Watanabe J., Shimizu Y., Ino-Oka E., Takishima T. (1983). Effects of afterload reduction on global left ventricular and regional myocardial functions in the isolated canine heart with stenosis of a coronary arterial branch. Circulation.

[35] Haines D.D., Lekli I., Teissier P., Bak I., Tosaki A. (2012). Role of haeme oxygenase-1 in resolution of oxidative stress-related pathologies: Focus on cardiovascular, lung, neurological and kidney disorders. Acta Physiol..

[36] Kupai K., Szabo R., Veszelka M., Awar A.A., Torok S., Csonka A., Barath Z., Posa A., Varga C. (2015). Consequences of exercising on ischemia-reperfusion injury in type 2 diabetic Goto-Kakizaki rat hearts: Role of the HO/NOS system. Diabetol. Metab. Syndr..

[37] Posa A., Kupai K., Menesi R., Szalai Z., Szabo R., Pinter Z., Palfi G., Gyongyosi M., Berko A., Pavo I. (2013). Sexual dimorphism of cardiovascular ischemia susceptibility is mediated by heme oxygenase. Oxid. Med. Cell. Longev..

[38] Posa A., Pavo I., Varga C. (2015). Heme oxygenase contributes to estradiol and raloxifene-induced vasorelaxation in estrogen deficiency. Int. J. Cardiol..

[39] Danial N.N., Korsmeyer S.J. (2004). Cell death: Critical control points. Cell.

[40] Maslov L.N., Naryzhnaia N.V., Podoksenov Iu K., Prokudina E.S., Gorbunov A.S., Zhang I., Pei Zh M. (2015). Reactive oxygen species are triggers and mediators of an increase in cardiac tolerance to impact of ischemia-reperfusion. Rossiiskii fiziologicheskii zhurnal imeni IM Sechenova.

[41] Mao G.D., Thomas P.D., Lopaschuk G.D., Poznansky M.J. (1993). Superoxide dismutase (SOD)-catalase conjugates. Role of hydrogen peroxide and the fenton reaction in sod toxicity. J. Biol. Chem..

[42] Bagatini M.D., Martins C.C., Battisti V., Gasparetto D., da Rosa C.S., Spanevello R.M., Ahmed M., Schmatz R., Schetinger M.R., Morsch V.M. (2011). Oxidative stress versus antioxidant defenses in patients with acute myocardial infarction. Heart Vessels.

[43] Stange E., Agostini B., Paenberg J. (1975). Changes in rabbit lipoprotein properties by dietary cholesterol, and saturated and polyunsaturated fats. Atherosclerosis.

[44] Cohen E., Aviram M., Khatib S., Volkova N., Vaya J. (2016). Human carotid atherosclerotic plaque protein(s) change HDL protein(s) composition and impair HDL anti-oxidant activity. BioFactors.

[45] Kim E.J., Kim B.H., Seo H.S., Lee Y.J., Kim H.H., Son H.H., Choi M.H. (2014). Cholesterol-induced non-alcoholic fatty liver disease and atherosclerosis aggravated by systemic inflammation. PLoS ONE.

[46] Hui C., Like W., Yan F., Tian X., Qiuyan W., Lifeng H. (2010). *S*-Allyl-l-cysteine sulfoxide inhibits tumor necrosis factor-α induced monocyte adhesion and intercellular cell adhesion molecule-1 expression in human umbilical vein endothelial cells. Anat. Rec..

[47] Kertesz A., Bombicz M., Priksz D., Balla J., Balla G., Gesztelyi R., Varga B., Haines D.D., Tosaki A., Juhasz B. (2013). Adverse impact of diet-induced hypercholesterolemia on cardiovascular tissue homeostasis in a rabbit model: Time-dependent changes in cardiac parameters. Int. J. Mol. Sci..

[48] Kovacs A., Fulop G.A., Kovacs A., Csipo T., Bodi B., Priksz D., Juhasz B., Beke L., Hendrik Z., Mehes G. (2016). Renin overexpression leads to increased titin-based stiffness contributing to diastolic dysfunction in hypertensive mRen2 rats. Am. J. Physiol. Heart Circ. Physiol..

[49] Juhasz B., Varga B., Czompa A., Bak I., Lekli I., Gesztelyi R., Zsuga J., Kemeny-Beke A., Antal M., Szendrei L. (2011). Postischemic cardiac recovery in heme oxygenase-1 transgenic ischemic/reperfused mouse myocardium. J. Cell. Mol. Med..

